# The Role of Sialic Acids in the Establishment of Infections by Pathogens, With Special Focus on *Leishmania*

**DOI:** 10.3389/fcimb.2021.671913

**Published:** 2021-05-13

**Authors:** Tainá Cavalcante, Mariana Medina Medeiros, Simon Ngao Mule, Giuseppe Palmisano, Beatriz Simonsen Stolf

**Affiliations:** ^1^ Laboratory of Leishmaniasis, Department of Parasitology, Institute of Biomedical Sciences, University of Sao Paulo, Sao Paulo, Brazil; ^2^ GlycoProteomics Laboratory, Department of Parasitology, Institute of Biomedical Sciences, University of Sao Paulo, Sao Paulo, Brazil

**Keywords:** *Leishmania*, sialic acids, infection, host-pathogen, Siglec

## Abstract

Carbohydrates or glycans are ubiquitous components of the cell surface which play crucial biological and structural roles. Sialic acids (Sias) are nine-carbon atoms sugars usually present as terminal residues of glycoproteins and glycolipids on the cell surface or secreted. They have important roles in cellular communication and also in infection and survival of pathogens. More than 20 pathogens can synthesize or capture Sias from their hosts and incorporate them into their own glycoconjugates and derivatives. Sialylation of pathogens’ glycoconjugates may be crucial for survival inside the host for numerous reasons. The role of Sias in protozoa such as *Trypanosoma* and *Leishmania* was demonstrated in previous studies. This review highlights the importance of Sias in several pathogenic infections, focusing on *Leishmania*. We describe in detail the contributions of Sias, Siglecs (sialic acid binding Ig-like lectins) and Neuraminidase 1 (NEU 1) in the course of *Leishmania* infection. A detailed view on the structural and functional diversity of *Leishmania*-related Sias and host-cell receptors will be provided, as well as the results of functional studies performed with different *Leishmania* species.

## Introduction

### Carbohydrate Roles in Host-Pathogen Interaction

The term ‘host–pathogen interaction’ relates to the many ways a pathogen interacts with its host (Casadevall and Pirofski, 1999; Sen et al., 2016). Research on host-pathogen interactions is an important and complex field, especially because new pathogens are frequently discovered, and each of them may have specific mechanisms of interaction with its host (Sen et al., 2016; Casadevall and Fang, 2020). Apart from the basic aspects of studying pathogen biology, understanding details of the interactions between the two or more species may help in disease prevention and cure. In fact, the study of such interactions will help understanding the pathogen´s biology, how its entry point into the host is facilitated and how it survives inside its host (Sen et al., 2016).

Pathogens may be transmitted by vectors, by ingestion, congenitally, by contact or by inhalation. For intracellular pathogens, further entry in cells is initiated by the recognition of host cell surfaces, and subsequent adhesion is essential for invasion or internalization (Imberty and Varrot, 2008). The survival of internalized pathogens then depends on their ability to reduce or resist immune responses of the host cells, such as the production of cytokines and nitric oxide (NO) (Sen et al., 2016).

Carbohydrates or glycans are ubiquitous components of the cell surface. They play crucial biological and structural roles including establishing both protective physical barriers against the outside environment, participating in cell–cell and cell–extracellular matrix interactions and regulation of intracellular signaling (Varki and Schauer, 2009; Huang et al., 2016). The structural variability and complexity of cell surface glycans improves their function as signaling, recognition and adhesion molecules (Ghazarian et al., 2011). Membranes of vertebrates´ cells are covered by a dense layer of glycoconjugates that forms the glycocalyx, comprising glycoproteins and glycolipids (Varki, 2017). The glycocalyx serves as an initial and important contact for pathogens (Karlsson, 2001). In fact, a diversity of viral, bacterial, and protozoan pathogens recognizes carbohydrates on cell surfaces to engage, colonize, and infect their hosts (Huang et al., 2016).

Apart from the host cell, many pathogens are also covered by a glycocalyx. For example, *Leishmania* protozoan parasite surface molecules include lipophosphoglycans (LPGs), glycoinositolphospholipids (GIPLS), proteophosphoglycans (PPG) and the 63 kDa glycoprotein gp63, which are anchored to the membrane by glycophosphoinositol (GPI) glycolipids (Ilgoutz and McConville, 2001). These macromolecules are pivotal in host-pathogen interaction, playing essential roles including escape from the hosts immune surveillance, host cell recognition, binding and invasion, and modulation of the host immune system (Ilg, 2000; Ilgoutz and McConville, 2001; Gupta et al., 2013; Mule et al., 2020). Furthermore, the role of glycans as vaccine targets has also been demonstrated (Russell and Alexander, 1988; Pinheiro et al., 2007). In BALB/c mice, protection against *L. mexicana* infection has been demonstrated using promastigote secretory gel (PSG) and a chemically defined synthetic glycovaccine synthesized with glycan structures similar to *L. mexicana* (Rogers et al., 2006).

Microorganisms like fungi, bacteria and protozoa have Sias and its derivatives as the major components of their glycocalyx (Vimr and Lichtensteiger, 2002; Alves et al., 2017). The frequent position of Sias in the terminal residues of glycoproteins and glycolipids favors the participation of this molecule in several biological processes, including cell-cell interaction (Crocker and Varki, 2001; Schauer and Kamerling, 2018).

### Sialic Acid Types and Diversity

**Figure 1:**
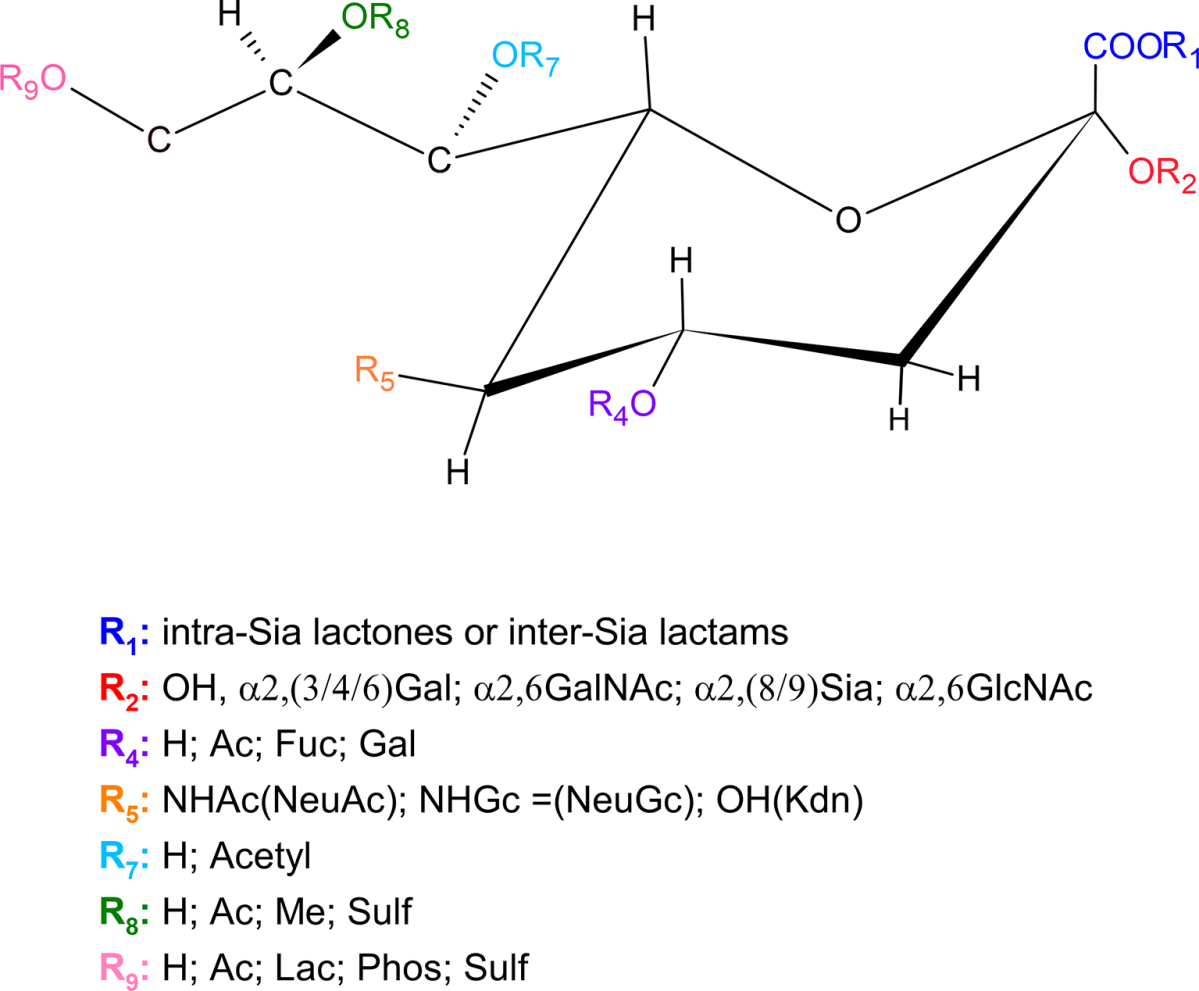
Sialic acid chemical structure and its possible variations. Ac, Acetyl; Fuc, Fucose; Gal, Galactose; GalNAc, *N*-acetylgalactosamine; GlcNAc, *N*-Acetylglucosamine; H, Hydrogen; Kdn, 2-Keto-3-deoxynonulosonic-acid; Lac, Lactose; Me, Methyl; NHAc, *N*-Acetylneuraminic acid; OH, Hydroxyl; Phos, Phosphate; Sulf, Sulfate (Traving and Schauer, 1998; Thaysen-Andersen et al., 2013; Schauer and Kamerling, 2018).

Sias are monosaccharides usually present as terminal residues of glycoproteins and glycolipids, either on the cell surface or secreted (Brinkman-Van der Linden and Varki, 2000). They are acidic nine-carbon atoms sugars derivatives of the basic neuraminic acid molecule (Neu), 5-amino-3,5-dideoxy-2- nonulosonic acid (Schauer et al., 2011) (**Figure 1**). More than 60 different Sias are known, which differ in terms of structural modifications of the sugar (Angata and Varki, 2002; Varki and Schauer, 2009).

The huge diversity of sialylated glycoconjugates results mainly from two factors. The first relates to the different α linkages between the C2 of Sias and sugars, catalyzed by different sialyltransferases (STs). The most common linkages occur at C3 or C6 positions of galactose or at C6 position of *N*-acetylgalactosamine, but Sias may also be found attached to other Sias mainly at C8 position (Angata and Varki, 2002; Varki and Schauer, 2009). The second source of diversity of sialylated glycoconjugates relates to natural modifications. In neuraminic acid (Neu), the 5-amino group is not *N*-acylated. More commonly, C5 position has an *N*-acetyl group (generating Neu5Ac) or a hydroxyl group (as in Kdn). The 5-*N*-acetyl group may be further hydroxylated, generating *N*-glycolylneuraminic acid (Neu5Gc). Human normal tissues do not contain NeuGc, and this sugar is immunogenic in humans (Kawano et al., 1994). The four core Sia molecules mentioned (Neu5Ac, Neu5Gc, Kdn, and Neu) may additionally have one or more substitutions at the hydroxyl groups on C4, C7, C8, and C9 (*O*-acetyl, *O*-methyl, *O*-sulfate, *O*-lactyl, or phosphate groups) (Varki and Schauer, 2009). Besides, although the C1 carboxylate group is usually ionized at physiological pH, it can be turned into a lactone with hydroxyl groups of adjacent saccharides or into a lactam (Varki and Schauer, 2009). Unsaturated and anhydro forms of free Sias also exist; of which “2-deoxy2,3 didehydroNeu5Ac” (Neu2en5Ac) is the most common. The combination of the above-mentioned glycosidic linkages with all the possible modifications is responsible for generating the vast diversity of known Sias (Varki and Schauer, 2009).

The high abundance of Sias on cell surface, lysosomal membranes and secreted glycoproteins reinforces their roles in the stabilization of molecules and membranes and in interactions with other cells or molecules (Varki and Schauer, 2009). The common terminal position of Sias favors its role of regulating biological processes involving cell-cell and cell-extracellular matrix interactions by either directly interacting with specific surface receptors or by masking other carbohydrate recognition sites (Kelm and Schauer, 1997; Schauer, 2000; Angata and Varki, 2002). Sias may also occur in internal positions in the distal end of glycan chains, the most common being one Sia residue attached to another (Angata and Varki, 2002). Internal Sias are also found in the repeating units of some bacterial polysaccharides and echinodermal oligosaccharides (Varki and Schauer, 2009).

### The Role of Sialyltransferases and Sialidases

Sialyltransferases (STs) catalyze the addition of Sias from activated Sias donor, cytidine-5′-monophosphate-Neu5Ac, to the terminal position of glycoconjugates, *N*- and *O*-linked glycoproteins and/or glycolipids, forming α-sialosides (Harduin-Lepers et al., 2005; Datta, 2009; Audry et al., 2011). According to their linkage and glycoconjugate acceptor, STs can be classified in four families. In vertebrates, twenty STs have been described being divided into: six α2,6-sialyltransferases (ST6GalNAcI-VI) that transfer Sia (Audry et al., 2011) to the hydroxyl group in C6 of the GalNac in *O*-linked glycoproteins (ST6GalNAc-I, II and IV) or glycoplipids (ST6GalNAc-III, V and VI). Another two α2,6-sialyltransferases (ST6GalI and II) transfer Sias as described above using as acceptor the terminal galactose. Six α2,3-sialyltransferases (ST3GalI-VI) catalyze the α2,3-linkage between Neu5Ac and C3 of terminal galactose residues found on glycoproteins and glycolipids. Moreover, six STs (ST8Sia-I to VI) transfer Neu5Ac to the hydroxyl group in C8 of another terminal Neu5Ac residue (Harduin-Lepers et al., 2005; Teppa et al., 2016). Beside sialyltransferases, mammals also have sialidases.

Sialidases/Neuraminidases are glycosidases that catalyze the removal of α-glycosidically linked Sia residues in glycoproteins and glycolipids (Miyagi and Yamaguchi, 2012). These enzymes can be found in vertebrates and microorganisms, including protozoa. Until now, four types of human sialidases have been identified and characterized, named NEU 1, NEU 2, NEU 3 and NEU 4 (Miyagi and Yamaguchi, 2012). Each sialidase is expressed in a specific tissue in a species-specific manner. In human tissues, NEU 1 usually has the highest abundance (10–20 times higher than other sialidases), followed by NEU 3 and NEU 4. On the other hand, NEU 2 abundance is extremely low or undetectable in normal human tissue (Hata et al., 2008; Nath et al., 2018).

The combinatorial expression of STs and NEUs dictates the profile of sialoglycans present on cell surfaces. A list of sialyltransferases and sialidases, as well as their targets and locations, is shown in **Table 1**.

**Table 1:** STs and sialidases - functions, targets, and locations (Harduin-Lepers, 2010; Audry et al., 2011; Miyagi and Yamaguchi, 2012; Glanz et al., 2019).

Sialyltransferases	Functions	Targets/Substrates	Locations
Sialidases			
**ST6GalNAc-I to VI**	Transfers Sia to the hydroxyl group in C6 of the GalNac in *O*-linked glycoproteins (ST6GalNAc-I, II, IV) or glycolipids (ST6GalNAc-III, V, VI)	*O-*glycoproteins (ST6GalNac-I, II) *O-*glycoproteins/glycolipids (ST6GalNac-III, IV) Glycoproteins/glycolipids (ST6GalNac-V) Glycolipids (ST6GalNac-VI)	Golgi apparatus
**ST6Gal-I and II**	Transfers Sia to the hydroxyl group in C6 of a terminal galactose residue	*N*-glycoproteins (ST6Gal-I, II)	Golgi apparatus
**ST3Gal-I to VI **	Catalyzes the α2,3-linkage between Neu5Ac and C3 of terminal galactose residues found on glycoproteins and glycolipids	*O-*glycoproteins/glycolipids (ST3Gal-I and II) Glycoproteins (ST3Gal-III) Glycoproteins/glycolipids (ST3Gal-IV and VI) Glycolipids (ST3Gal-V)	Golgi apparatus
**ST8Sia-I to VI**	Transfers Neu5Ac to the hydroxyl group in C8 of another terminal Neu5Ac residue	Glycolipids (ST8Sia-I, V) Glycoproteins (ST8Sia-II, IV) Glycolipids/glycoproteins (ST8Sia-III) *O*-glycoproteins (ST8Sia-VI)	Golgi apparatus
**NEU 1**	Phagocytosis Elastic fiber assembly Exocytosis Immune responses Lysosome catabolism	Oligosaccharides Glycopeptides	Lysosomes
**NEU 2**	Neural cells and myoblast differentiation	Oligosaccharides Glycoproteins Gangliosides	Cytosol and plasma membrane
**NEU 3**	Adhesion Neural cells differentiation Apoptosis	Gangliosides	Plasma membrane
**NEU 4**	Adhesion Neural cells differentiation Apoptosis	Oligosaccharides Glycoproteins Gangliosides	Lysosomes, mitochondria and endoplasmic reticulum

### The Relevance of Sias to Pathogens

More than 20 pathogens can synthesize or capture Sias from their hosts and incorporate them into their own glycoconjugates (Crocker et al., 2007; Severi et al., 2007; Khatua et al., 2010). Sialylation of pathogens´ glycoconjugates may be crucial for their survival for several reasons. They may mimic host cell surfaces, leading to evasion of immune attack, may reduce interactions with host cells by electrostatic repulsion, as observed for negatively charged Sias, and may inhibit activation of the alternative pathway of complement (although the opposite has already been observed, as will be discussed later). Besides, pathogen Sias may promote infection by their binding to host surface receptors and by their modulation of immune response (Crocker et al., 2007; Khatua et al., 2010; Khatua et al., 2013; Roy and Mandal, 2016). Indeed, Sias on surface glycoconjugates of pathogens are commonly involved in interaction with host cells and thus affect disease progression (Crocker and Varki, 2001; Mukhopadhyay and Mandal, 2006; Bandyopadhyay and Mandal, 2008; Ghoshal et al., 2010; Khatua et al., 2010; Campetella et al., 2020).

Pathogens exploit their own Sias and also host Sias for establishing infection and survival. For example, many viruses recognize and bind to Sias linked to glycoproteins and gangliosides of the host cell. This interaction is essential for viruses such as influenza, parainfluenza, mumps, corona, norovirus, rotavirus, and DNA tumor viruses (Matrosovich et al., 2013). Pathogenic bacteria also make use of Sias for their benefit. They use Sias as nutrient and also coat themselves with the molecule, avoiding phagocytosis and the lytic action of the complement cascade (Severi et al., 2007). The ability of *V. cholerae* to take up host Sias confers advantages in intestinal colonization (McDonald et al., 2016). Besides, bacteria such as enterohemorrhagic *Escherichia coli*, *Haemophilus influenzae*, *Haemophilus ducreyi*, *Neisseria gonorrhoeae* and *Neisseria meningitidis* overlay their surfaces with Sias, hiding their antigens from the host immune system (Almagro-Moreno and Boyd, 2009).

Apart from its roles in viral and bacterial infection, Sia also plays important roles in protozoan diseases. Merozoites of *Plasmodium falciparum*, etiological agent of malaria, use Sia dependent and Sia independent mechanisms for erythrocyte invasion (Cowman and Crabb, 2006; Ord et al., 2012). Sia dependent mechanisms rely on the binding of *Plasmodium* proteins to glycophorins, the major sialylated proteins on the erythrocyte (Jungery et al., 1983).

*Toxoplasma gondii*, the protozoan parasite responsible for toxoplasmosis, also recognizes Sias in the host cells, and the attachment to these molecules is essential for invasion. In fact, the amount of intracellular parasites is proportional to the abundance of Sias in host cell surface, being low for cells with low or no surface Sias and high for cells in which Sias were artificially added using *Trypanosoma cruzi* trans-sialidase (Monteiro et al., 1998). A *T. gondii* Sias binding protein named SABP1 (Sialic Acid Binding Protein) was recently identified in a proteomic analysis of Sias-high-affinity binding proteins (Xing et al., 2020). Parasites knockout for SABP1 showed lower adhesion and infection *in vitro* and lower virulence *in vivo*, indicating the importance of SABP1-Sias interaction for parasite survival and disease establishment (Xing et al., 2020).

### Sias in Trypanosomatids

Trypanosomatids refer to the group of flagellated protozoans that belong to the Trypanosomatidae family. This family comprises 12 different genera, and parasites from the genera *Trypanosoma* and *Leishmania* are etiological agents of important human diseases (Souto-Padron, 2002). The surface of trypanosomatids is characterized by a negative net charge, conferred mainly by Sias associated with glycoproteins, glycolipids and phosphate groups (Souto-Padron, 2002). Due to their negative charges, Sias can inhibit some interactions but can also serve as ligands for different receptors (Varki, 1997).

Sias participate in several steps of *Trypanosoma cruzi* interaction with its host. Glycosylation in *T. cruzi* cell surface was described long ago by agglutination of epimastigote stage parasites to concanavalin A (Con A) lectin (Alves and Colli, 1974). Later, the decoration of *T. cruzi* surface by Sias was demonstrated by agglutination assays using wheat germ agglutinin (WGA) lectin (Pereira et al., 1980), and two Sias, Neu5Ac and Neu5Gc, were characterized on *T. cruzi* epimastigotes (Schauer et al., 1983). The biosynthetic pathway for Sias incorporation was identified, indicating an alternative pathway for the incorporation of Sias to *T. cruzi* glycoconjugates by an unusual trans-glycosylase activity (Previato et al., 1985). Indeed, *T. cruzi* cannot synthesize Sias, but it captures the molecule from host’s sialylglycoconjugates by its cell surface and secreted trans-sialidase (TS) activity (Schenkman et al., 1991; Serrano et al., 1995; Abuin et al., 1996; Ferguson, 1997; Alves and Colli, 2008; Campetella et al., 2020). Interestingly, *T. cruzi* TS increases in the parasite’s infective stage, reinforcing its role in virulence (Campetella et al., 2020). A recent study concluded that TS shed to the extracellular milieu, and not TS anchored to the membrane, are responsible for the sialylation of mucins (Lantos et al., 2016). In contrast to earlier observations, this study also showed that TS are shed in microvesicles, and not in a soluble form by hydrolysis of TS GPI-anchors (Lantos et al., 2016). A family of *O*-glycosylated, threonine-rich mucin-like glycoproteins are the main acceptors of Sias in *T. cruzi* surface. Mucins are the most abundant molecules in *T. cruzi*, and the main components of the parasite´s glycocalyx (Freire-de-Lima et al., 2012). They play important roles in the invasion of the host and subversion of its immune system (Morrot, 2013).

Despite the participation of *T.cruzi* surface Sias on the interaction with cells, the role of these molecules on host cell infection by this parasite is still controversial. While many studies point that surface Sia contributes to adhesion and infection (Schenkman et al., 1991; Campetella et al., 2020), some suggest that the molecule is not essential or even that it compromises host cell invasion (revised in (Freire-de-Lima et al., 2012). Anyway, it has been shown that sialylation increases parasite´s resistance to killing by lytic antibodies and also hampers the activation of the complement cascade (Kipnis et al., 1981; Freire-de-Lima et al., 2012; Morrot, 2013). Besides, sialylated glycoconjugates modulate the dendritic cell function and decrease T cell activation and proliferation in response to mitogens and antigens (Morrot, 2013). The effect of Sias on CD4+ T cells is mediated by sialic acid binding Immunoglobulin-like lectins (Siglecs), especially Siglec-E. This receptor is expressed mostly in mouse phagocytic cells and on antigen-presenting cells (APCs) including macrophages and dendritic cells (Morrot, 2013).

In *Trypanosoma brucei*, the conversion from bloodstream trypomastigotes to insect procyclic trypomastigotes is accompanied by an increase in the expression of a TS that shares several characteristics with the *T. cruzi* enzyme (Pontes de Carvalho et al., 1993). Moreover, Sias scavenged from the host allows *T. brucei* parasites to survive in Tsetse fly (Nagamune et al., 2004). Procyclic trypomastigotes are covered by procyclin, which is the main acceptor of Sias in the parasite (Pontes de Carvalho et al., 1993; Montagna et al., 2002). Furthermore, the characterization of procyclin glycosylphosphatidylinositol membrane anchor of *T. brucei* showed that it’s highly decorated with Sias (Ferguson et al., 1993).

Analysis of *Crithidia luciliae* and *Crithidia fasciculata* surface showed that phosphate groups and Sias significantly contributed to the negative surface charge of these organisms (Motta et al., 1991; Matta et al., 1995).

The presence of Sias was also demonstrated in several species of *Leishmania*, the causative agents of leishmaniasis. The characteristics of *Leishmania* Sias, the process of Sias acquisition and the roles of these molecules in parasite binding to host cells and in immune system evasion will be discussed in the following topics.

### Sias in Different *Leishmania* Species

*Leishmania* parasites belong to Trypanosomatidae family, and several species have been described as causative agents of leishmaniasis. Leishmaniasis is a serious and widespread disease, responsible for around 12 million cases worldwide.

The life cycle of *Leishmania* includes the extracellular promastigote stage transmitted to the vertebrate host by the insect vector. Promastigotes do not invade cells, but are internalized by several phagocytic cells, mainly macrophages (Kaye and Scott, 2011). The macrophage receptors involved in the recognition and phagocytosis of parasites are mainly complement (CR), mannose, fibronectin and Fc-γ receptors (Ueno and Wilson, 2012). Inside the macrophage, promastigote converts into the amastigote form, which proliferates inside the phagolysosome (Podinovskaia and Descoteaux, 2015).

*Leishmania* promastigotes are covered by a dense glycocalyx composed of glycosylphosphatidylinositol-anchored glycoproteins, glycoinositol-phospholipids, lipophosphoglycans, glycolipids, and proteoglycans (Ilg et al., 1992; Ilg et al., 1994; Guha-Niyogi et al., 2001; Mule et al., 2020). These molecules are known to participate in many processes throughout the parasite’s life cycle and are key players in disease establishment and pathogenesis (Guha-Niyogi et al., 2001; Kamhawi, 2006).

Sias were described in promastigotes and amastigotes of several *Leishmania* species (Chatterjee et al., 2003; Chava et al., 2004; Ghoshal et al., 2010). Due to its position in terminal residues of glycoconjugates, Sias participate on the interaction between *Leishmania* and host cell receptors (Karmakar et al., 2012).

In *Leishmania*, Sias form α2→3 and α2→6 linkages with sugar residues of the parasite membrane, being α2→6 the most frequent type of linkage (Chatterjee et al., 2003; Ghoshal et al., 2010). *Leishmania* Sias can further be *O*-acetylated generating 9-*O*-acetylated (9-*O*-AcSA) Sias (Bandyopadhyay and Mandal, 2008; Varki and Schauer, 2009). The predominance of α2→6 links was evident in *L. tropica*, *L. major*, *L. braziliensis* and *L. infantum* and was less pronounced in *L. amazonensis* and *L. mexicana*. Sias was also described in *L. donovani* promastigotes and amastigotes, in which predominate α2→3, α2→6 and 9-*O*-acetylated types (Chatterjee et al., 2003; Chava et al., 2004; Ghoshal et al., 2010). The predominance of α2-6-linked Sias in *Leishmania* promastigotes was demonstrated by different analytical and biochemical methods (Ghoshal et al., 2010).

The abundance of Sias varies according to the *Leishmania* species. Comparative analysis of the virulent *Leishmania* strains *L. tropica* K27, *L. major* JISH118, *L. braziliensis* L280, *L. infantum* MON29, *L. mexicana* LV4 and *L. amazonensis* LV81 indicated different total Sias levels, despite their similarities in pathogenicity. In fact, K27, JISH118, L280 and MON29 showed high Sias (“Sias-high strains”) and enhanced proportion of 9-*O*-acetyl Sias (percentage of Sias 9-*O*-acetylated), whereas LV4 and LV81 had reduced Sias (“Sias-low strains”) and low proportion of 9-*O*-acetyl Sias (Ghoshal et al., 2010). These virulent strains have higher Sias compared to an avirulent *L. donovani* strain (UR6) strain (Ghoshal and Mandal, 2011). Sias levels in the virulent strains correlated with intracellular survival and resistance to nitric oxide, and α2-6-linked Sias were proportionally more abundant in “Sias-high strains” (Ghoshal et al., 2010). Besides, enzymatic removal of *O*-acetyl and of Sias from promastigotes led to a decrease in phagocytosis and *in vitro* infection (Ghoshal and Mandal, 2011).

The types of Sias and their affinities to Siglec receptors also vary among *Leishmania* strains (Ghoshal et al., 2010). Sias may also vary between parasite life stages. Indeed, *L. donovani* amastigotes exhibit another derivative of Sias, *N*-glycolylneuraminic acid (Neu5Gc), never described in promastigotes (Chava et al., 2004; Ghoshal and Mandal, 2011).

The results described indicate that Sia types and abundance vary among *Leishmania* strains and species, and that this molecule contributes to parasite virulence, affecting phagocytosis and infection. The methods employed to study *Leishmania* Sias are described in **Table 2**, as well as representative references that employed them.

**Table 2:** Methods employed for analysis and characterization of *Leishmania* Sias.

Methods	*Leishmania* species/strain	Main objectives/findings	References
Fluorimetric quantification by acetyl acetone method	*L.tropica* K27, *L. major* JISH118, *L. mexicana* LV4, *L. braziliensis* L280, *L. amazonensis* LV81, *L. infantum* MON29	Quantity of total Sias is proportional to virulence	(Ghoshal et al., 2010)
HPLC	K27, LV4, LV81	Different proportions of Neu5Gc, Neu5Ac, Neu5,7Ac2 and other derivatives	(Chatterjee et al., 2003; Chava et al., 2004; Ghoshal et al., 2010; Karmakar et al., 2012)
ELISA	K27, JISH118, L280, MON29, LV4, LV81, and *L. donovani* AG83	Sias correlation with host responses	(Chatterjee et al., 2003; Ghoshal et al., 2010)
Lectin blot (Western blot)-employing labeled achatinin	MHOM/IN/83/AG83	Identification of Sias on amastigotes and adsorbed serum sialoglycans on promastigotes	(Chatterjee et al., 2003; Chava et al., 2004)
Lectin blot (Western blot)- MAA e SNA labeled lectins	K27, JISH118, L280, MON29, LV4, LV81, AG83	The role of Sias in *Leishmania* infection	(Chatterjee et al., 2003; Chava et al., 2004; Ghoshal et al., 2010; Karmakar et al., 2012)
Flow cytometry employing lectins conjugated with fluorophores	K27, JISH118, L280, MON29, LV4, LV81, AG83	Sias and the host susceptibility response	(Chava et al., 2004; Ghoshal et al., 2010; Karmakar et al., 2012)

### Acquisition and Metabolism of Sias in *Leishmania*

The two ways by which microbes obtain Sia are by *de novo* biosynthesis and by acquisition from the environment (Lauc and Heffer-Lauc, 2006). *Trypanosoma cruzi* acquires Sias by transglycosylation, employing a cell surface and secreted TS (and not an intracellular CMP-Sia dependent transferase) to add Sias to glycoconjugates (Previato et al., 1985; Colli, 1993; Pereira-Chioccola and Schenkman, 1999), as mentioned. A machinery similar to the one described in *T.cruzi* has not been reported in *Leishmania* (Buschiazzo et al., 2000; Karmakar et al., 2012).

Initial findings indicated that *Leishmania* promastigotes incorporate sialoglycans from the fetal calf serum present in the cell culture medium and probably did not have pathways for *de novo* biosynthesis (Chatterjee et al., 2003). To verify if gangliosides and sialoglycoproteins from serum could be used by promastigotes to produce Sias, desialylated parasites were incubated with different sialoglycans in serum-free medium. The results indicated that exogenous sialoglycans could indeed be used for *de novo* glycoprotein sialylation by *Leishmania* promastigotes (Karmakar et al., 2012).

The biosynthesis of sialoglycans is a complex process involving CMP-Sia, CMP-Sia transporter and sialyltransferases (Jacobs et al., 2001). Golgi sialyltransferases must encounter the glycoconjugate substrate and CMP-Sia, which must be transported to the Golgi by a CMP-Sia transporter (Zhao et al., 2006). The analysis of *L. infantum* genome revealed two putative genes at chromosome 24 coding for Sias transporters, which are homologous to genes found in other *Leishmania* species (Karmakar et al., 2012). Apart from transporters, both α2,3-sialyltransferase and α2,6-sialyltransferase activities were observed in *Leishmania* (Karmakar et al., 2012). Besides, the two enzymes were detected by immunofluorescence mainly in the Golgi of the parasites, employing antibodies for human sialyltransferases that cross react with *Leishmania* transferases. A higher activity of α2,3-sialyltransferasewas noted in virulent *L. donovani* parasites (Karmakar et al., 2012).

### Siglecs

Siglecs are type 1 transmembrane proteins and are the best characterized I-type lectins. Siglecs are composed of an amino-terminal V-set Ig-like domain, variable numbers (1 to 16) of C2-set Ig-like domains, a single-pass transmembrane domain and a cytosolic tail of variable length (Crocker et al., 2007). The terminal V domain is responsible for binding to Sias, which seems to be the only ligand to these receptors (Angata et al., 2004; Crocker et al., 2007) (Crocker and Varki, 2001). Siglecs have different specificities for Sias, which are determined by the highly variable C–C´ loop of the V-set domain (Crocker et al., 2007). The number of C2-set Ig-like domains of each Siglec determines its interaction with Sias on the same cell surface (cis-interaction) or on adjacent cells (trans-interaction) (Bornhofft et al., 2018).

Siglecs were discovered simultaneously in macrophages and B cells and were later shown to be present mostly in hematopoietic cells, although also expressed in some cells outside the immune system (Varki and Angata, 2006; Crocker et al., 2007). Fourteen different Siglecs were already described in humans and nine in mice, which are organized in two groups based on their sequence similarities and homologies. One group includes Siglec-3 (CD33)-related Siglecs, while the other comprises Siglec-1 (Sialoadhesin or CD169), Siglec-2 (CD22), Siglec-4 (MAG) and Siglec-15 (Angata et al., 2004; Crocker et al., 2007).

Recognition by Siglecs plays important roles in the immune system, and may either activate or inhibit the immune response, depending on the presence of ‘immune receptor tyrosine-based activation motif’ (ITAM) or ‘immune receptor tyrosine-based inhibition motifs’ (ITIM) on their cytoplasmic region, respectively (Bornhofft et al., 2018). Two Siglec receptors, Siglec-1 and Siglec-4, have no ITIMs or ITAMs (Bornhofft et al., 2018). Apart from directly activating and inhibiting signalling, Siglecs also have roles in cell-cell interaction (Crocker et al., 2007), as will be discussed later.

In the last ten years, different studies have demonstrated the importance of the interaction between macrophage Siglecs 1 and 5 and *Leishmania* Sias in parasite phagocytosis and intracellular survival (Karmakar et al., 2012; Roy and Mandal, 2016; Van Bockstal et al., 2020). Many reviews describe details on Siglecs’ structures and functions (Crocker and Varki, 2001; Crocker et al., 2007; Klaas and Crocker, 2012; Bornhofft et al., 2018), thus, this review will focus mainly on Siglec 1 and 5, the Sias receptors already described to participate in *Leishmania* infection.

Siglec-1, CD169 or sialoadhesin (Sn), is one of the largest molecules of Ig superfamily, with 17 extracellular domains (Crocker et al., 1994). It has preferential binding to α2–3-linked over α2–6-linked Sias, and lower affinity to α2–8-linked Sias (Brinkman-Van der Linden and Varki, 2000).

Siglec-1 was first described in bone marrow ‘stromal’ macrophages involved in erythropoiesis (Crocker and Gordon, 1985). It was then shown to be a marker of a macrophage subpopulation present in bone marrow, lymph node, liver and spleen (Crocker et al., 2007; Klaas and Crocker, 2012; Chavez-Galan et al., 2015).

Siglec-1 positive macrophages were shown to be involved in immune tolerance and antigen presentation and are capable of binding to erythrocytes (Chavez-Galan et al., 2015). As mentioned earlier, Siglec-1 lacks tyrosine-based signaling motifs. Besides, its cytoplasmic tail is not very conserved, suggesting a primary role in cell-cell interactions instead of signaling (Crocker et al., 2007). It is constitutively present on the surface of some subpopulations of tissue-resident macrophages, but rapidly increases after inflammatory stimuli, pointing to a pro-inflammatory function in macrophages (Crocker et al., 2007). This induction agrees with the observation that Siglec-1 is an interferon stimulated gene product (York et al., 2007). Models of different inflammatory and infectious diseases suggest that Siglec-1 modulates T-cell function and activation. Besides, it may act as a phagocytic receptor to sialylated pathogens (Crocker et al., 2007). In fact, most Sia-dependent binding to macrophages rely on Siglec-1, suggesting that it is the major macrophage Sia receptor involved in cell-cell interactions (Klaas and Crocker, 2012).

Human and mouse Siglec-1 proteins have 72% sequence identity (Hartnell et al., 2001). Siglec-1 deficient mice have very mild alterations when kept in pathogen-free conditions, suggesting that the receptor has no roles in immune cell development (Oetke et al., 2006). They have less B220-positive B cells and more CD8 T cells in spleens and lymph nodes, but no differences in cell composition of bone marrow, peritoneal cavity, and thymus (Oetke et al., 2006). These results suggest that Siglec-1 may regulate immune cell function but not steady-state hematopoiesis (Oetke et al., 2006).

Siglec-5 is an inhibitory receptor that contains an ITIM domain, and phosphorylation of tyrosine residue in ITIM generates docking sites for SHP-1 and/or SHP-2 containing phosphatases (Karmakar et al., 2012). These phosphatases become activated and dephosphorylate proteins that regulate cellular activation. SHPs negatively regulate nitric oxide (NO) production and inhibit MAPKs (mitogen activated protein kinase) and Akt signaling pathways (Nandan et al., 1999). These two pathways lead to Nuclear factor κβ (NF-κβ) activation, inducing the expression of pro-inflammatory genes (Zha et al., 2014).

### Siglec’s Roles in *Leishmania* Infection

Macrophage Siglecs were shown to be important for binding to *L. donovani* promastigotes (Roy and Mandal, 2016), but only a few studies analyze functional roles of Sias and Siglec in *Leishmania* infection. Siglec-1 participates in phagocytosis and infection, contributing to the parasite’s engulfment by the macrophage*. L. donovani* “Sias-high” promastigotes were shown to preferentially bind to CHO cells expressing Siglec-1 and Siglec-5. Accordingly, virulent parasites with abundant Sias bind more to macrophages through Siglec-1 and Siglec-5 compared to low Sias avirulent parasites (Roy and Mandal, 2016). Besides, removal of Sias, Siglec-1 blocking by neutralizing antibody and Siglec-1 reduction by siRNA knockdown led to reduced *L. donovani* binding to macrophages (Karmakar et al., 2012; Roy and Mandal, 2016).

The importance of Siglec-1 in infections by *Leishmania* was also supported by the increase in intracellular parasites after stimulation with interferon-α (IFN-α). Indeed, IFN-α induces upregulation in surface Siglec-1, which correlates with increase in macrophage infection by *L. infantum* and *L. donovani* (Van Bockstal et al., 2020).

Apart from binding to *Leishmania*, macrophage Siglecs also participate in the signaling triggered by the parasite (Roy and Mandal, 2016). As previously mentioned, the binding of Sias to Siglec-5 increases activation of macrophage SHP-1. Increase of SHP-1 in macrophages infected with Sias-high *L. donovani* reinforced the role of parasite Sias in upregulation of this phosphatase (Abu-Dayyeh et al., 2008; Roy and Mandal, 2016). Besides, infection led to an increase in the association of Siglec-5 and SHP-1 (Roy and Mandal, 2016).

*Leishmania* infection is known to inhibit macrophage MAPK signaling (Ben-Othman et al., 2008). Accordingly, removal of *L. donovani* Sias with neuraminidase or reduction of macrophage Siglec-5 levels with siRNA decreased phosphatase activity and reversed parasite-induced inhibition of MAPK signaling (Karmakar et al., 2012).

**Figure 2:**
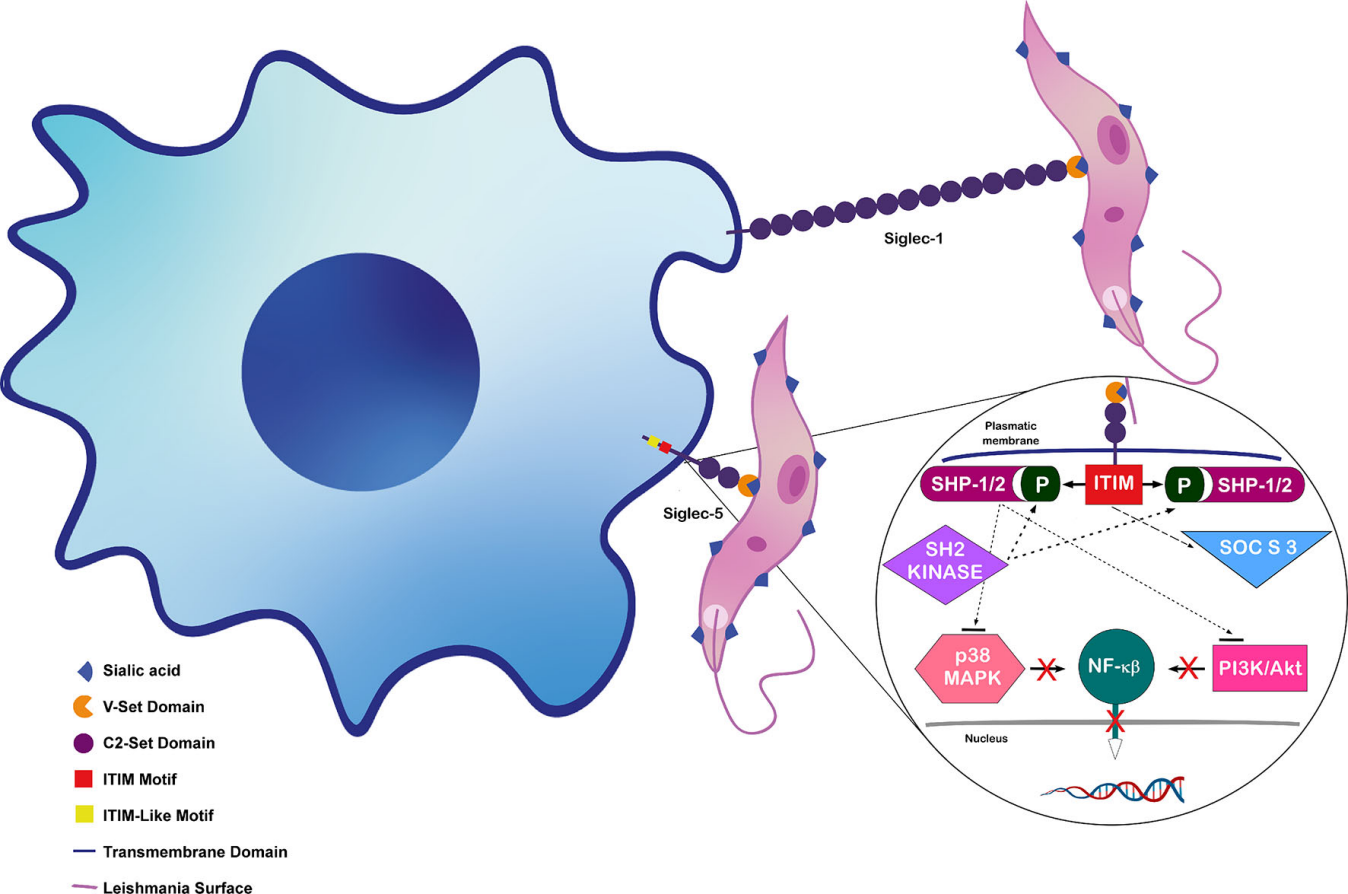
Interaction between *Leishmania donovani* Sias and macrophage Siglecs. Siglec-1 binding to *L. donovani* surface Sias enhances entry on macrophage and Siglec-5 binding downregulates host cell signaling pathway (shown in detail). Figure adapted from (Khatua et al., 2013, Siddiqui et al., 2019), inspired in data and structures from (Crocker et al., 2007; Karmakar et al., 2012; Roy and Mandal, 2016; Siddiqui et al., 2019).

These studies indicate that Sias-Siglec interactions are important in parasite binding to macrophage and intracellular signaling. **Figure 2** shows the binding of *Leishmania* Sias to macrophage Siglecs 1 and 5 and the pathways induced after Sias-Siglec-5 interaction.

### Sias and Immune Response in Leishmaniasis

Gamma interferon (IFN-γ) and interleukin 4 (IL-4) are key players both in visceral and in cutaneous leishmaniasis. Visceral leishmaniasis (VL) is usually accompanied by a systemic immunosuppression manifested by decreased levels of IFN-γ and IL-12, along with an increased Th2 response, illustrated by elevated levels of IL-4 and IL-10 (Zwingenberger et al., 1990; Ghalib et al., 1993; Bacellar et al., 1996). This profile results from the reduction of expression of Th1 cytokine genes (IFNγ and IL-2) and upregulation of Th2 cytokine genes (IL-4, IL-10 and TGFβ). IL-10 is also considered a very potent immunosuppressive cytokine in VL pathogenesis (Mondal et al., 2010).

Sias-mediated interaction between *Leishmania* and macrophages *in vitro* usually results in dominant Th2 type cytokine response (high IL-10, IL-4 and TGFß) and lower macrophage ROS and NO levels, resulting in enhanced intracellular amastigote survival and replication (Roy and Mandal, 2016). The impairment of Sias-Siglec interaction by removal of parasite Sias or reduction/silencing of Siglec from macrophages results in a decrease in Th2 response and in parasite burden (Roy and Mandal, 2016).

Sias-mediated interactions during *L. donovani* infection also induce the suppression of PI3K, p-PDK1 and pAkt in PI3K/Akt pathway (Roy and Mandal, 2016). Infection with desialylated *L. donovani* led to the reversal of phosphorylation of these molecules, indicating a role of parasite Sias on deactivation of this pathway (Roy and Mandal, 2016). The involvement of *Leishmania* Sias in the regulation of the translocation of a functional subunit of NF-κβ to the nucleus was also demonstrated. *L. donovani* infection reduced the abundance of inhibitor of NF-κβ kinase (IKK) β and α in the macrophage cytoplasm, leading to a reduction in IKKα and IKKβ-mediated phosphorylation and degradation of inhibitory subunit IκBα. The presence of IκBα complexed with NF-κβ in the cytosol avoided NF-κβ translocation to the nucleus and transcription of inflammation-related genes (Roy and Mandal, 2016).

Apart from the effect of 9-*O*-AcSA in NO production, this Sia also confers resistance to this oxide (Ghoshal et al., 2010). In fact, 9-*O-*AcSA high *Leishmania* showed higher survival and multiplication in human macrophages when compared to their deacetylated forms, and induced lower levels of NO, interleukin-12 and interferon-γ (Ghoshal et al., 2010). Since inhibition of NO production by macrophages correlates with increased survival of intracellular parasites and severity of the disease, Sias can be associated with an evasion mechanism for *Leishmania*.

Patients with VL show alteration not only in Th1 x Th2 balance but also in other cellular aspects. For example, they have more 9-*O*-acetylated sialoglycans (9-*O*-AcSGs) with α2-6 linkage on their hematopoietic cells, as well as higher levels of antibodies against these molecules (Sharma et al., 1998; Bandyopadhyay et al., 2004). These antibodies were shown to induce complement deposition and lysis of red blood cells and of *Leishmania* promastigotes (Bandyopadhyay et al., 2004). Patients with VL also display disease-associated 9-*O*-acetylated sialoglycoproteins on their peripheral blood mononuclear cells (PBMC), which decrease after parasitological cure (Ghoshal et al., 2009). The presence of these molecules modulates the immune system towards the Th1 profile, providing a good correlation between patient´s 9*-O-*AcSGPs and the control of parasitemia and disease pathology (Ghoshal et al., 2009).

Sialylation of host receptors also affects *Leishmania* infection. A recent study showed the importance of the lysosomal sialidase NEU 1 in macrophage Toll like receptor 4 (TLR4) desialylation and its role in *L. donovani* infection (Karmakar et al., 2019).

Macrophages participate on innate immunity by recognizing pathogen-associated molecular patterns through Toll-like receptors (TLRs). Myeloid differentiation factor 88 (MyD88) associates with most TLRs and participates in downstream events that lead to translocation of nuclear factor κB (NF-κB) (Sugiyama et al., 2016). NF-κB induces the expression of several pro-inflammatory genes, including those encoding cytokines and chemokines, and also participates in inflammasome regulation (Liu et al., 2017).

**Figure 3:**
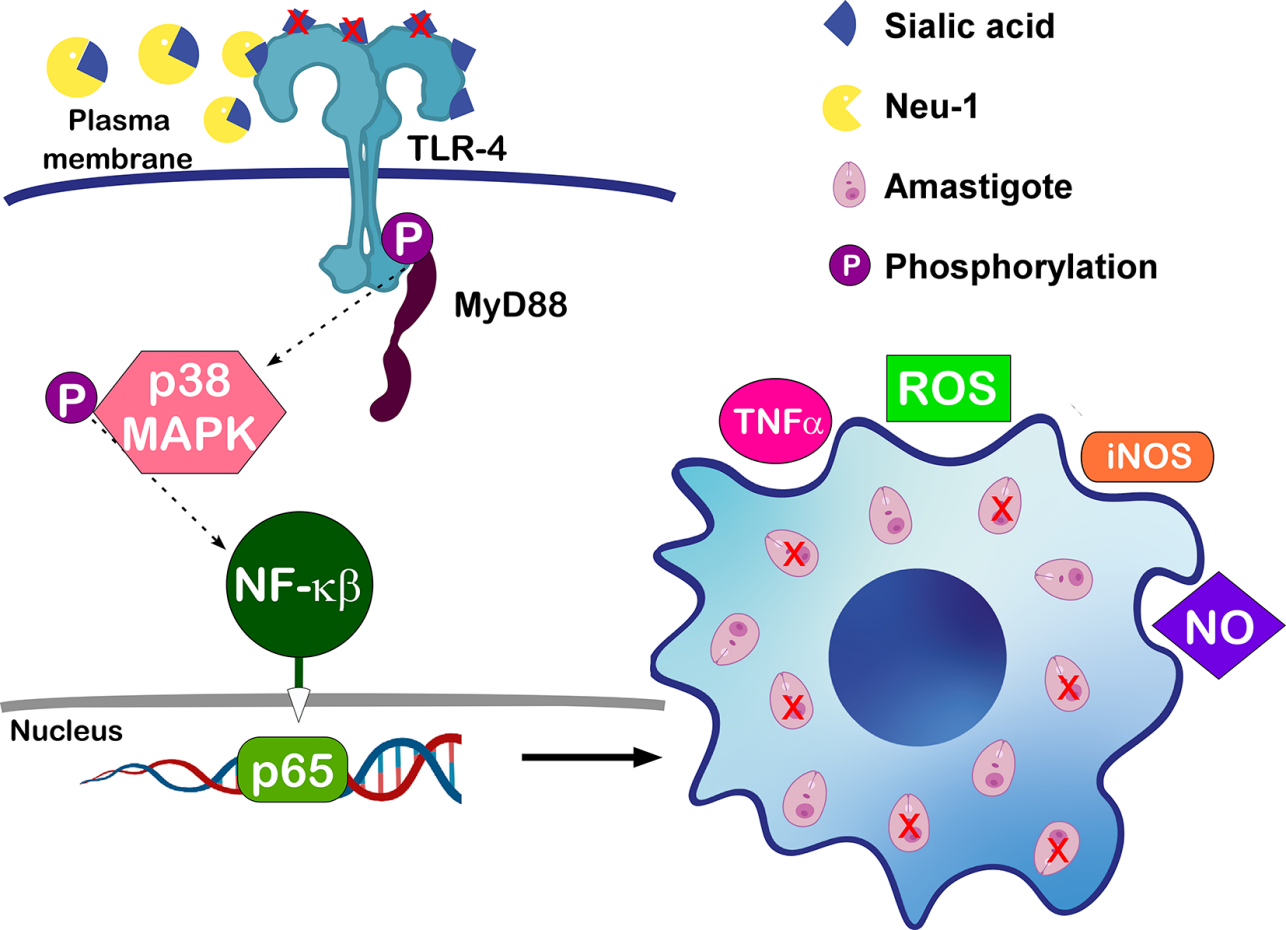
Effects of NEU 1 in macrophages´ infection by *L. donovani*: Desialylation of TLR4 by NEU 1 increases receptor´s association with MyD88 and activation of MAPK pathway. Activation leads to nuclear translocation of p-65 and upregulation of proinflammatory cytokines and other mediators in infected cells, leading to parasite death (Karmakar et al., 2019).

The expression of NEU 1 is upregulated during monocyte to macrophage differentiation, increasing the phagocytic capacity of the latter cells (Liang et al., 2006). Desialylation of sialyl α-2,3-linked β-galactosyl residues of macrophage TLR4 by NEU 1 seems to be important for receptor activation and cellular signaling *via* NF-κβ (Amith et al., 2010). In fact, TLR4 was shown to be necessary for efficient parasite control, leading to NO synthesis and *Leishmania* death (Tuon et al., 2008; Miyagi and Yamaguchi, 2012). Accordingly, the above-mentioned work showed that infection of macrophages with *L. donovani* reduced the association of NEU1 with TLR4, increasing TLR4 sialylation. The higher receptor´s sialylation resulted in a reduced association with the downstream adaptor protein MyD88, a lower activation of MAP kinase signaling pathway and impaired innate immune activation (Karmakar et al., 2019). These observations were validated by NEU 1 overexpression in macrophages followed by *L. donovani* infection, which caused enhanced association of TLR4 with NEU 1 and of TLR4 with MyD88, leading to increased levels of Th1 cytokines and NO secretion, reducing parasite burden, as illustrated in **Figure 3**.

Taken together, the findings mentioned here highlight that not only parasite but also host molecules’ sialylation affect infections by *Leishmania*.

## Discussion

Sias are essential molecules for either unicellular or complex multicellular organisms. They play roles in cellular communication and also in infection and survival, in the case of pathogens.

This review highlights the importance of Sias in several pathogenic infections and provides details on the roles of Sias and their receptors Siglec 1 and 5 in *Leishmania* infection and disease pathology. The abundance and the types of Sias vary according to parasite species and strains, and several studies showed association of higher concentrations of Sias and increased infection. The recognition of Sias by Siglecs not only increases parasite entrance but also affects macrophage signaling and immune response. In fact, the level of sialylation of TRL4, regulated by its association with NEU 1, impacts on its activation and in parasite death.

There are several studies focused on the characterization of *Leishmania* Sias, but very few focused on the functional aspects or roles of these molecules on infection. Besides, most studies analyzed only one or a few species, mostly *L. donovani*, and were conducted by the same research group or by related groups. *L. donovani* was also the only species analyzed in functional studies. The heterogeneity observed among *Leishmania* species in terms of molecules and virulence mechanisms points to the need for further research of Sias contribution to pathogenicity of different species and of human isolates of the parasite.

A better understanding of the complexity of *Leishmania* Sias, the pathways involved in their production by the parasite and their recognition by macrophage receptors may help in the identification of possible targets for a safe specific treatment for leishmaniasis.

## Author Contributions

All authors contributed to the final version of the manuscript. BS organized the manuscript structure and topics. TC and MMM wrote most topics. TC prepared the figure. SM wrote specific topics. BS and GP revised the whole manuscript. All authors contributed to the article and approved the submitted version.

## Funding

This work was supported by FAPESP grants (BSS: 2018/14972-8 and GP: 2018/18257-1, 2018/15549-1), and by CAPES (TC), CNPq (MM) and FAPESP (SM, 2017/04032-5) scholarships.

## Conflict of Interest

The authors declare that the research was conducted in the absence of any commercial or financial relationships that could be construed as a potential conflict of interest.
